# Cerebral autoregulation in anoxic brain injury patients treated with targeted temperature management

**DOI:** 10.1186/s40560-021-00579-z

**Published:** 2021-10-26

**Authors:** Ilaria Alice Crippa, Jean-Louis Vincent, Federica Zama Cavicchi, Selene Pozzebon, Filippo Annoni, Antonella Cotoia, Hassane Njimi, Nicolas Gaspard, Jacques Creteur, Fabio Silvio Taccone

**Affiliations:** 1 Department of Intensive Care, Erasme Hospital, Université Libre de Bruxelles (ULB), Route de Lennik 808, 1070 Bruxelles, Belgium; 2 Department of Anesthesia and Intensive Care, University Hospital of Foggia, Viale Luigi Pinto 1, 71122 Foggia, Italy; 3 Department of Neurology, Erasme Hospital, Université Libre de Bruxelles (ULB), Route de Lennik 808, 1070 Bruxelles, Belgium

**Keywords:** Post-anoxic brain injury, Neurological outcome, Temperature management

## Abstract

**Background:**

Little is known about the prevalence of altered CAR in anoxic brain injury and the association with patients’ outcome. We aimed at investigating CAR in cardiac arrest survivors treated by targeted temperature management and its association to outcome.

**Methods:**

Retrospective analysis of prospectively collected data. Inclusion criteria: adult cardiac arrest survivors treated by targeted temperature management (TTM). Exclusion criteria: trauma; sepsis, intoxication; acute intra-cranial disease; history of supra-aortic vascular disease; severe hemodynamic instability; cardiac output mechanical support; arterial carbon dioxide partial pressure (PaCO_2_) > 60 mmHg; arrhythmias; lack of acoustic window. Middle cerebral artery flow velocitiy (FV) was assessed by transcranial Doppler (TCD) once during hypothermia (HT) and once during normothermia (NT). FV and blood pressure (BP) were recorded simultaneously and Mxa calculated (MATLAB). Mxa is the Pearson correlation coefficient between FV and BP. Mxa > 0.3 defined altered CAR. Survival was assessed at hospital discharge. Cerebral Performance Category (CPC) 3–5 assessed 3 months after CA defined unfavorable neurological outcome (UO).

**Results:**

We included 50 patients (Jan 2015–Dec 2018). All patients had out-of-hospital cardiac arrest, 24 (48%) had initial shockable rhythm. Time to return of spontaneous circulation was 20 [10–35] min. HT (core body temperature 33.7 [33.2–34] °C) lasted for 24 [23–28] h, followed by rewarming and NT (core body temperature: 36.9 [36.6–37.4] °C). Thirty-one (62%) patients did not survive at hospital discharge and 36 (72%) had UO. Mxa was lower during HT than during NT (0.33 [0.11–0.58] vs. 0.58 [0.30–0.83]; *p* = 0.03). During HT, Mxa did not differ between outcome groups. During NT, Mxa was higher in patients with UO than others (0.63 [0.43–0.83] vs. 0.31 [− 0.01–0.67]; *p* = 0.03). Mxa differed among CPC values at NT (*p* = 0.03). Specifically, CPC 2 group had lower Mxa than CPC 3 and 5 groups. At multivariate analysis, initial non-shockable rhythm, high Mxa during NT and highly malignant electroencephalography pattern (HMp) were associated with in-hospital mortality; high Mxa during NT and HMp were associated with UO.

**Conclusions:**

CAR is frequently altered in cardiac arrest survivors treated by TTM. Altered CAR during normothermia was independently associated with poor outcome.

**Supplementary Information:**

The online version contains supplementary material available at 10.1186/s40560-021-00579-z.

## Introduction

Mortality and morbidity remain high after cardiac arrest (CA) [1]. Although survival rate has improved over the years [2], less than 10% of patients recover an intact neurological function [3]. The pathophysiology of hypoxic–ischemic brain injury involves microvascular dysfunction, micro-thrombosis, vasogenic and cytotoxic edema, neuronal cell dysfunction and excitoxicity [4–6]. Reduction of cerebral blood flow (CBF) in the presence of arterial hypotension has been suggested as a potential contributor to secondary damage [7, 8], although CBF assessment is rarely performed in clinical practice.

Current guidelines underline the importance of post-resuscitation care in preventing secondary brain injury. Targeted temperature management (TTM) remains the only neuroprotective strategy, although its effectiveness remains controversial [9]. Maintaining an adequate CBF and cerebral oxygen delivery appears an appealing strategy [10]. Although it is recommended to keep mean arterial blood pressure (MAP) above 65 mmHg, such target may not guarantee an adequate cerebral perfusion in CA patients. In fact, pressure-cerebral autoregulation (CAR), which is an active mechanisms that maintains a constant CBF through changes in the diameter of cerebral arterioles within a wide range (i.e., 50–150 mmHg) of MAP in healthy individuals [11], might be altered after an acute illness [12–14]. As such, changes in MAP would lead to directly proportional modifications in CBF, with the risk of either cerebral hypoperfusion or hyperemia. Altered CAR has been reported in CA patients [7, 8, 15–17] and associated with poor outcome [8, 15]. However, some of these studies were conducted before of the TTM era and differed in methods to assess CAR.

The aim of this study was to investigate changes in CAR over time in patients treated with TTM after CA. Secondarily, we aimed to assess the prognostic role of CAR on patients’ outcome.

## Methods

*Study population* Data of patients admitted to our Department of Intensive Care after CA were included into a prospective registry (approved by the Ethics Committee, who waived informed consent—P2015/394). CAR was assessed routinely in brain-injured patients by use of transcranial Doppler (TCD), except in case of: traumatic brain injury (TBI); sepsis, drug intoxication; acute intra-cranial disease; previous intra or extra-cranial vascular surgery; MAP < 50 mmHg; extra corporeal membrane oxygenation, intra-aortic balloon pump counter-pulsation; arterial carbon dioxide partial pressure (PaCO_2_) > 60 mmHg; cardiac arrhythmias; lack of acoustic window. This study (P2018/234) retrospectively included all patients > 18 years admitted from January 2015 to December 2018, treated by TTM and with available CAR data.

*Patients’ management* Patients unresponsive after return of spontaneous circulation (ROSC), without contraindication, underwent induction of hypothermia (HT, core temperature 32–34 °C) for at least 24 h with subsequent rewarming not faster than 0.25 °C per hour. Following rewarming, core body temperature > 38 °C was treated. Patients were mechanically ventilated; sedation and neuro-muscular blocking agents (NMBA) were provided as needed. Invasive arterial blood pressure (BP) was monitored in radial or femoral artery. Temperature was invasively monitored (pulmonary artery catheter, PiCCO^®^). All patients had continuous electroencephalogram (cEEG) monitoring according to the International 10 to 20 system as soon as possible after admission, for at least 48 h and as long as clinically necessary.

*TCD.* TCD was performed once during HT and once during normothermia (NT, core body temperature > 36.5 °C). Left or right middle cerebral artery (MCA) was identified [18] with a 2-MHz probe, which was held in place using a plastic device (DWL, Germany. Blood flow velocity (FV) in MCA and BP were recorded simultaneously (DWL, Germany). TCD was performed when the patient was in steady state condition, avoiding any stimuli, modifications of vasoactive drugs, sedation, fluidic therapy or ventilator settings, 14 [9–18] h (HT) and 38 [36–45] h (NT) from admission. Arterial blood gas analysis (ABG), use of sedatives and NMBA, respiratory setting and use of cardio-active medications (i.e., inotropic agents or vasopressors) were recorded. cEEG were reviewed by a neurophysiologist (NG) and classified according to the American Clinical Neurophysiology Society definitions [19]. If suppressed or burst-suppression background were observed anytime during the monitoring, cEEG was classified as “highly malignant pattern” (HMp) [20, 21]. The highest value of neuron-specific enolase (NSE) over the first 72 h, since ICU admission was recorded. Survival was assessed at hospital discharge. Cerebral Performance Category scale (CPC) 3 months after CA was recorded from follow-up consultations. Unfavorable neurological outcome (UO) was defined as CPC 3–5, favorable outcome (FO) as CPC 1–2.

*Cerebral autoregulation analysis* FV and BP recordings were downloaded on a personal computer. Artifacts were identified as FV and BP values exceeding 3 standard deviations (MATLAB, USA) and visually inspected. In case of artefacts, the entire cardiac cycle was discarded. Recordings were artifacts were > 20% were discarded from analysis. CAR was assessed using Mxa index [22]; Mxa is the Pearson’s correlation coefficient between BP and FV, averaged on a 10 s moving window with 50% overlap. Mxa > 0.3 defined altered CAR [12].

*Study outcomes* The primary outcome was the differences in Mxa values and the proportion of patients with altered CAR between UO and FO. Secondary outcomes included: the differences in Mxa values and the proportion of patients with altered CAR between survivors and non-survivors; the predictive role of Mxa for hospital mortality and UO; the association between Mxa, highly malignant cEEG and NSE values.

*Statistical analysis* Statistical analysis was performed using R statistical software version 4.0.3 (R Foundation for Statistical Computing), Prism (GraphPad Software Inc.) and SAS software (SAS Institute Inc.). Characteristics of population are described as median [IQRs] or counts (%). Shapiro–Wilk test was used to assess normality of data. Data were compared using Mann–Whitney test, Wilcoxon rank test, Kruskal–Wallis test or Fisher Exact test as appropriate. Difference in Mxa values among CPC groups was tested by one-way ANOVA with Tukey post hoc analysis. Correlation between Mxa and NSE was assessed using Spearman coefficient. In the multivariable analyses, considering the limited number of events, the predictor variables that were highly collinear within and across outcome and the risk of overfitting, we constructed predictive models for each outcome using tenfold cross validation with penalized logistic regression [23] (providing odds ratio and 95% confidence intervals) and, as a sensitivity analysis, using generalized linear model via regularized regression with elastic net. Elastic net regression is controlled by two parameters, (1) alpha, which sets the degree of mixing between two extremes of regularized regression, and (2) lambda, defining the strength of regularization [24]. Linearity of the continuous variables with respect to the logit of the dependent variable was assessed via the Box–Tidwell procedure. Presence of outliers were assessed by studentized residuals. Collinearity between variables was assessed by variance inflation factor. All test are two tailed and the statistical significance was set at the 5% level.

## Results

### Study population

Out of 194 patients admitted over the study period, 50 were available for the analysis and 144 were excluded (*n* = 12 not treated with TTM; *n* = 4 TBI or acute intracranial disease; *n* = 13 previous stroke or neurological disease; *n* = 10 hemodynamic instability: *n* = 22 ECMO; *n* = 8 elevated PaCO_2_; *n* = 28 cardiac arrhythmias, *n* = 18 lack of acoustic window; *n* = 29 lack of TCD operator). All patients had out-of-hospital CA. Shockable rhythm was recorded in 24 (48%) patients. Time to ROSC was 20 [10–35] min. HT (core temperature 33.7 [33.2–34] °C) lasted for 24 [23–28] h. During HT, all patients were sedated and 41 (82%) were on NMBA; during NT (core temperature 36.9 [36.6–37.4] °C), 25 (50%) patients were on sedation and 8 (16%) on NMBA. Thirty-one (62%) patients did not survive at hospital discharge. Of those, 23 (74%) died because of severe post-anoxic brain injury, while in 8 (26%) patients, complete prognostication was not completed and death occurred because of multiple organ failure. Overall, 36 patients (72%) had UO at 3 months: 5 (10%) patients had CPC 3 and 31 (62%) patients had CPC 5. Nine patients (18%) had full neurological recovery (CPC 1) and 5 patients (10%) had CPC 2.

### Cerebral autoregulation and outcomes

**Fig. 1:**
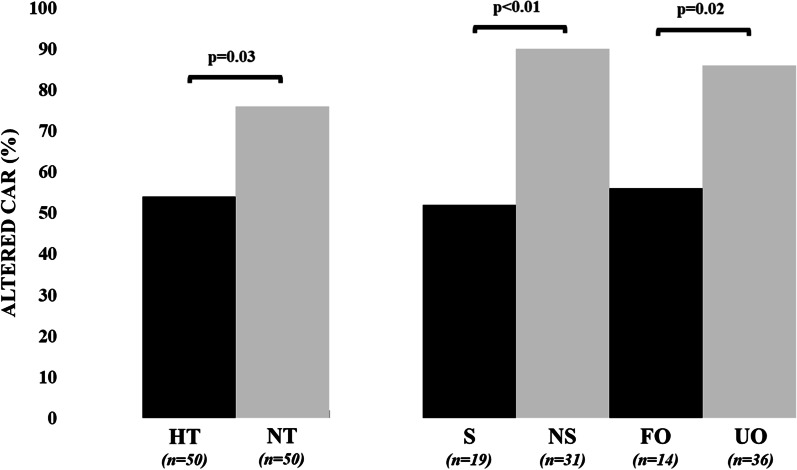
Proportion of patients with altered CAR, according to the phase of temperature control (HT = hypothermia; NT = normothermia) or the measured outcomes (S = survivors; NS = non-survivors; FO = favorable neurological outcome; UO = unfavorable neurological outcome). For outcome assessment, CAR was considered at NT

**Fig. 2:**
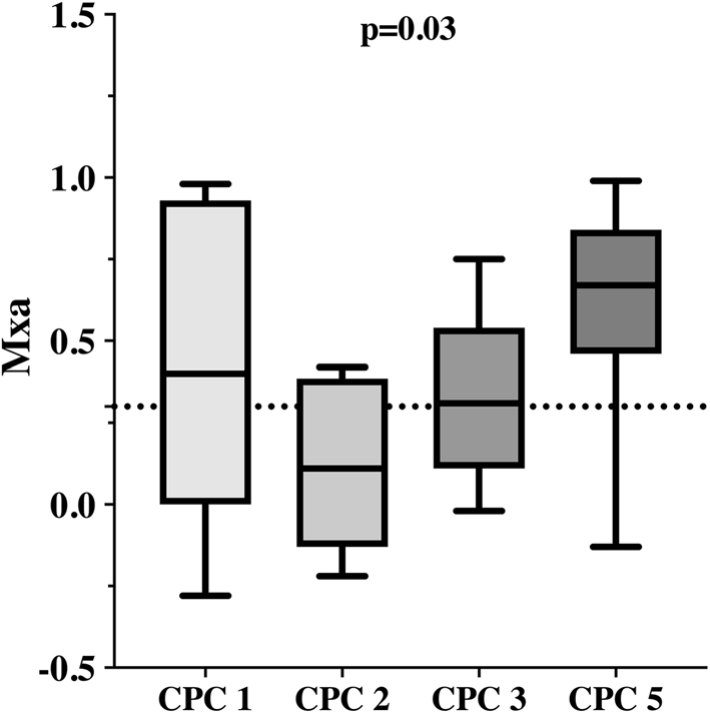
Mxa at NT, according to the different CPC. Mxa was 0.40 [0.00–0.92] in patients with CPC 1, 0.11 [− 0.13–0.39] in patients with CPC 2, 0.31 [0.17–0.43] in patients with CPC 3, 0.67 [0.46–0.84] in patients with CPC 5 (*p* < 0.01). CAR was altered in 5/9 (56%) patients with CPC 1, 2/5 (40%) patients with CPC 2, 3/5 (60%) patients with CPC 3 and 28/31 (90%) patients with CPC 5 *p* = 0.02)

Mxa was lower during HT than during NT (0.33 [0.11–0.58] vs. 0.58 [0.30–0.83]; *p* = 0.03—Additional file 1: Table S1). Altered CAR was more frequent during NT than during HT (76% vs. 54%; *p* = 0.03—Fig. 1). During HT, Mxa values and the proportion of patients with altered CAR were similar between patients with UO and FO (Table 1). During NT, patients with UO had higher Mxa values (0.63 [0.43–0.83] vs. 0.31 [− 0.01–0.67]; *p* = 0.03) and were more likely to have altered CAR compared to others (Table 1; Fig. 1). Values of clinical variables during normothermia in different outcome groups are reported in Table 2. Mxa values and the proportion of patients with altered CAR at NT differed among CPC scale values (*p* = 0.03 and *p* = 0.02, respectively—Fig. 2). CPC 2 had lower Mxa than CPC 3 and 5 groups. Four outliers in Mxa values were identified: one patient in the CPC 5 group had Mxa = − 0.40, three patients in CPC 1 group had Mxa = 0.90, 0.91 and 0.96, respectively. The outlier in the CPC 5 group with preserved CAR died because of MOF on high dose of cardio-active medication; 2 outliers in the CPC 1 group had PaCO_2_ between 50 and 60 mmHg, while the other had no clear explanation for high Mxa value. When Mxa analysis was performed excluding the first 3 outliers, patients with CPC 1, 2 and 3 had significantly lower Mxa values than CPC 5 subgroup (*p* < 0.01 for comparison between CPC 1–2 and CPC 5, *p* = 0.017 for comparison between CPC 3 and CPC 5—Additional file 1: Table S3).

**Table 1:** Characteristics of the study cohort according to outcomes

	All (*n* = 50)	Survivors (*n* = 19)	Non-Survivors (*n* = 31)	*p* value	FO (*n* = 14)	UO (*n* = 36)	*p* value
Male gender, *n* (%)	39 (78)	15 (79)	24 (77)	1.00	11 (79)	28 (79)	1.00
Age, years	65 [55–73]	60 [51–71]	67 [55–74]	0.33	58 [51–73]	66 [57–73]	0.40
APACHE II	26 [24–31]	26 [21–28]	27 [24–33]	0.07	26 [21–28]	27 [24–32]	0.17
ICU LOS, days	9 [4–19]	22 [11–37]	4 [3–10]	< 0.01	18 [9–31]	6 [3–13]	< 0.01
Comorbidities							
Arterial hypertension, *n* (%)	23 (46)	7 (37)	16 (52)	0.39	5 (36)	18 (50)	0.53
Vascular disease, *n* (%)	10 (20)	3 (16)	7 (23)	0.72	3 (21)	7 (19)	1.00
Chronic heart failure, *n* (%)	3 (6)	0 (0)	3 (10)	0.28	0 (0)	3 (8)	0.55
Chronic kidney disease, *n* (%)	8 (16)	2 (11)	6 (19)	0.69	1 (7)	7 (19)	0.41
COPD, *n* (%)	6 (12)	2 (11)	4 (13)	1.00	0 (0)	6 (17)	0.17
Diabetes mellitus, *n* (%)	10 (20)	3 (30)	7 (70)	0.72	1 (7)	9 (25)	0.25
Previous Seizure, *n* (%)	3 (6)	0 (0)	3 (10)	0.28	0 (0)	3 (8)	0.55
Minor stroke, *n* (%)	7 (14)	2 (11)	5 (16)	0.70	1 (7)	6 (17)	0.66
Cardiac arrest characteristics							
Cause of CA, *n* (%)							
* Cardiac*	23 (48)	13 (68)	10 (32)	0.02	10 (71)	13 (36)	0.03
* Anoxic*	15 (32)	2 (11)	13 (42)	0.03	1 (7)	14 (39)	0.04
* Obstructive*	2 (6)	1 (5)	1 (3)	1.00	1 (7)	1 (3)	0.49
* Indeterminate*	9 (18)	4 (22)	7 (10)	0.30	2 (14)	8 (26)	0.30
Shockable rhythm, *n* (%)	24 (48)	14 (58)	10 (42)	< 0.01	10 (71)	14 (39)	0.06
Time to ROSC, min	20 [10–35]	16 [10–35]	23 [16–35]	0.09	13 [10–33]	22 [16–36]	0.03
Clinical variables and autoregulation assessment							
NSE (ng/mL)	39 [24–78] (*n* = 32)	29 [20–40] (*n* = 14)	74 [34–108] (*n* = 18)	< 0.01	25 [20–37] (*n* = 10)	56 [32–86] (*n* = 22)	0.01
Highly malignant EEG	18 (36)	0 (0)	18 (58)	< 0.01	0 (0)	18 (50)	< 0.01
Mxa during HT	0.33 [0.11–0.58]	0.37 [0.03–0.51]	0.31 [0.18–0.65]	0.25	0.21 [0.00–0.46]	0.38 [0.18–0.65]	0.08
Mxa during NT	0.58 [0.30–0.83]	0.31 [0.00–0.62]	0.67 [0.46–0.84]	< 0.01	0.31 [− 0.01–0.69]	0.63 [0.43–0.83]	0.03
Altered CAR at HT, *n* (%)	27 (54)	11 (42)	17 (52)	0.77	7 (50)	20 (56)	0.76
Altered CAR at NT, *n* (%)	38 (76)	10 (53)	28 (90)	< 0.01	7 (50)	31 (86)	0.02

*APACHE II* acute physiology and chronic health evaluation II, *CA* cardiac arrest, *CAR* cerebral autoregulation, *COPD* chronic obstructive pulmonary disease, *FO* favorable outcome, *HT* hypothermia, *LOS* length of stay, *Mxa* mean flow index, *NS* non survivors, *NT* normothermia, *ROSC* return of spontaneous circulation; *S* survivors, *UO* unfavorable outcome

**Table 2:** Clinical and biological variables during the normothermic phase of TTM, according to outcomes

	Survivors (*n* = 19)	Non-survivors (*n* = 31)	*p* value	FO (*n* = 14)	UO (*n* = 36)	*p* value	
Sedatives, *n* (%)	6 (31)	19 (61)	0.08	4 (29)	21 (58)		0.11
NMBA, *n* (%)	1 (5)	7 (23)	0.13	1 (7)	7 (19)		0.41
Mechanical ventilation, *n* (%)	8 (42)	26 (84)	< 0.01	3 (21)	31 (86)		< 0.01
MAP, mmHg	77 [70–89]	75 [70–83]	0.47	81 [70–91]	75 [70–85]		0.37
Heart rate, bpm	91 [68–111]	88 [78–103]	0.82	93 [66–108]	88 [78–106]		0.65
Temperature, °C	37.0 [36.8–37.4]	36.8 [36.4–37.1]	0.08	37.1 [37.0–37.5]	36.8 [36.5–37.1]		0.04
Hemoglobin, g/dL	12.2 [10.7–14.0]	10.8 [8.9–12.8]	0.17	11.9 [10.5–14.0]	11.5 [9.3–13.7]		0.68
PEEP, cmH_2_O	5 [5–8]	6 [5–10]	0.46	5 [5–9]	6 [5–10]		0.51
FiO_2_	0.4 [0.3–0.5]	0.3 [0.3–0.5]	0.16	0.4 [0.3–0.5]	0.3 [0.3–0.5]		0.25
pH	7.36 [7.32–7.39]	7.40 [7.35–7.42]	0.20	7.38 [7.34–7.39]	7.4 [7.33–7.42]		0.88
PaCO_2_, mmHg	39 [37–40]	36 [34–40]	0.02	39 [37–42]	37 [34–40]		0.07
PaO_2_, mmHg	83 [70–92]	80 [72–91]	0.74	85 [76–94]	80 [71–91]		0.43
ScvO_2_, %	75 [72–79]	75 [73–78]	0.82	75 [72–79]	75 [72–78]		0.83
Lactate, mmol/L	1.3 [1.0–2.0]	1.3 [1.0–2.3]	0.66	1.3 [1.0–1.7]	1.3 [1.0–2.4]		0.37
Time to TCD assessment, hours	38 [35–43]	38 [36–46]	0.78	39 [36–42]	38 [32–48]		0.98
Cardio-active medications, *n* (%)	9 (47)	23 (74)	0.07	6 (43)	26 (72)		0.10

*FiO*_*2*_ fraction of inspired oxygen, *FO* favorable outcome, *MAP* mean arterial pressure, *NMBA* neuromuscular blocking agents, *PEEP* positive end-expiratory pressure, *PaCO*_*2*_ carbon dioxide arterial partial pressure, *PaO*_*2*_ oxygen arterial partial pressure, *ScvO*_*2*_ central venous oxygen saturation, *TCD* transcranial Doppler, *UO* unfavorable outcome

During HT, Mxa values and the proportion of patients with altered CAR were similar between non-survivors (NS) and survivors (SU) (Table 1). At NT, Mxa values were significantly higher in NS when compared to SU (0.67 [0.46–0.84] vs. 0.31 [0.00–0.62]; *p* < 0.01—Table 1) and altered CAR was observed more frequently in NS than SU (Table 1, Fig. 1).

**Fig. 3:**
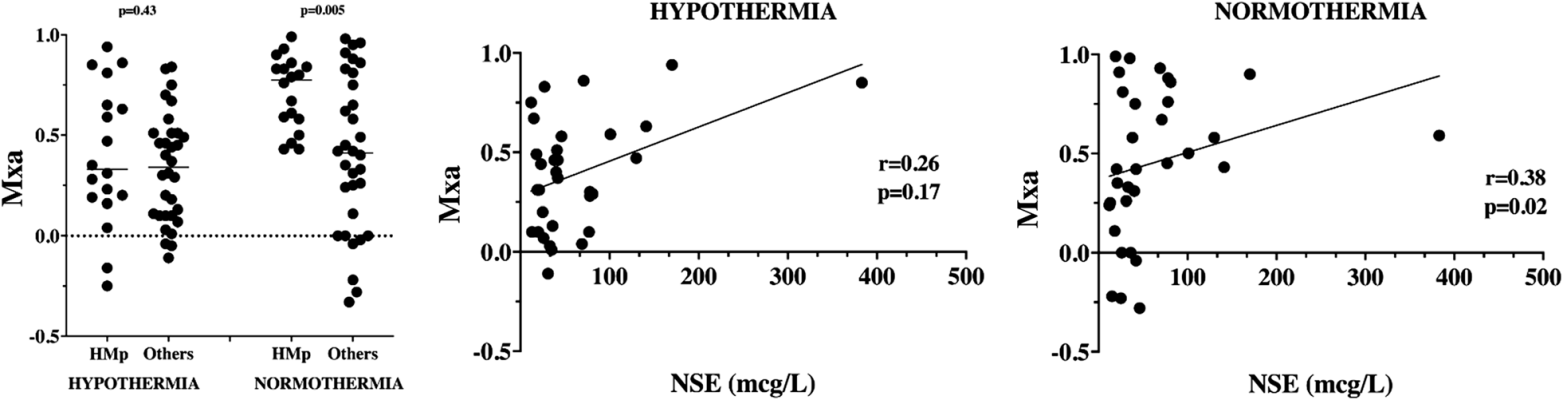
Mxa according to the presence of highly malignant cEEG pattern (HMp); correlation between Mxa and the highest neuron specific enolase (NSE) values at hypothermia and normothermia

Patients with highly malignant cEEG pattern (HMp) (*n* = 18) had higher Mxa values at NT than others (0.78 [0.56–0.85] vs. 0.41 [0.10–0.77]; *p* = 0.04). There was a correlation between NSE and Mxa at NT (*r* = 0.38 [95% CI 0.02–0.64]; *p* = 0.02) but not at HT (*r* = 0.26 [95% CI − 0.12–0.56]; *p* = 0.17—Fig. 3). Such results was confirmed when one very high value (= 383 ng/mL) was excluded from the analysis (at HT, *r* = 0.18; *p* = 0.32—at NT, *r* = 0.32; *p* = 0.04).

### Multivariable analyses

In the penalized logistic regression analysis, high Mxa during NT and HMp were associated with UO; the fitted model correctly classified 84% of patients (Sn 71.4%; Sp 97.2%). Using the same approach, initial non-shockable rhythm, high Mxa during NT and HMp were associated with in-hospital mortality (Table 3); the model correctly classified 81% of patients (Sn 68.4%; Sp 93.5%). Similar results were observed using regularized regression models with elastic net (Additional file 1: Table S4).

**Table 3:** Penalized logistic regression cross validation of predictive models

	Mortality		Unfavourable outcome	
	OR (CI)	*p* value	OR (CI)	*p* value
Age	1.04 (0.96–1.12)	0.347	1.02 (0.95–1.11)	0.550
Arterial hypertension	3.73 (0.69–20.16)	0.126	3.03 (0.63–14.64)	0.68
Shockable rhythm	0.15 (0.03–0.82)	0.029	0.44 (0.09–2.15)	0.312
Time to ROSC	1.01 (0.92–1.09)	0.909	1.01 (0.93–1.10)	0.809
Cardio-active medications	2.54 (0.47–13.75)	0.279	1.80 (0.37–8.74)	0.463
pCO_2_ (mmHg)	0.86 (0.72–1.02)	0.088	0.87 (0.73–1.05)	0.156
EEG HMp	16.42 (2.06–130.96)	0.008	10.01 (1.23–81.34)	0.031
Mxa	1.04 (1.01–1.07)	0.011	1.03 (1.01–1.06)	0.049

The fitted models showed an accuracy of 84% (CI 71–93%), a sensitivity of 68% and a specificity of 94% for mortality, and an accuracy of 86% (CI 73–94%), a sensitivity of 100% and a specificity of 50% for unfavourable outcome. *CI* confidence interval, *EEG* HMp highly malignant electroencephalography during ICU stay, *Mxa* mean flow index during normothermia, *OR* odds ratio, *pCO*_*2*_ arterial carbon dioxide partial pressure during normothermia, *ROSC* return of spontaneous circulation. Cardio-active medications: noradrenaline (norepinephrine) and/or dobutamine

## Discussion

This study showed that CAR is altered in CA patients treated by TTM, more often during normothermia than during hypothermia. Altered CAR during normothermia was more frequent among patients with poor outcomes. High Mxa values, thus worse CAR, were independently associated with hospital mortality and predictors of poor neurological recovery, such as highly malignant patterns on cEEG and high NSE values.

Our results are consistent with previous studies [7, 8, 15–17, 25]. Sundgreen et al. showed that CAR was altered in 8 out of 18 CA patients and the lower limit of CAR range was higher compared to healthy volunteers [7]. Ameloot et al. showed that altered CAR assessed by near infrared spectroscopy (NIRS) during the first 24 h of TTM was associated with poor neurological outcome and that MAP below the optimal CAR range was associated with mortality in 51 CA patients [8]. In another study (*n* = 23) body temperature was kept around 36 °C and altered CAR during the first 3 days after hospital admission was associated with mortality; however, CAR values did not change over time among survivors and non-survivors [15]. Similar CAR alteration was reported within 24 h from admission in 20 CA patients who underwent TTM at different target temperatures [25].

In this study, altered CAR after CA independently predicted poor outcome. However, whether altered CAR is a marker of brain injury or a contributing factor to outcome remains unclear; CAR was preserved early after CA, while altered in non-survivors and patients with UO at NT. Moreover, Mxa values at NT correlated with the highest NSE values during ICU stay, generally obtained 48–72 h after injury [26]. These findings suggest that altered CAR might result in altered cerebral perfusion inducing secondary brain damage in these patients. In previous studies, lower limit of CAR was right-shifted and the range of effective CAR narrowed after CA [7, 8, 15]. Furthermore, although the association between maintenance of MAP within CAR range and neurological recovery has not be determined [27], the amount of time spent at MAP outside CAR ranges has been associated with UO [8]. As MAP didn’t differ between HT and NT nor at NT between groups of patients with different outcomes, assessing autoregulation to individualize MAP values according to Mxa values rather than targeting predefined MAP targets (i.e., > 65 mmHg) may be beneficial in clinical settings.

The mechanisms underlying the impairment of CAR after CA are unclear, but involvement of cerebral vasculature is likely. During the first 12 h after CA, vascular resistances are often increased, while they decrease 24 h from resuscitation (i.e., secondary hyperemia) with CBF returning to normal values after 24 h from CA [28–30]. After 24 h from CA, low cerebrovascular resistances and high CBF have been associated with poor prognosis [31, 32]. In this study, abnormal Mxa values were more likely to be observed during NT. The vasodilation that accompanies the late hyperemic response could be responsible of the reduced vasoactive tone and loss of response to BP fluctuations, i.e., loss of CAR. Experimental studies suggest that moderate decrease in core body temperature might be associated to an extended range of CAR [33]. Lavinio et al. showed that rewarming was associated with altered CAR in 24 patients cooled for refractory intracranial hypertension following TBI [34]. An inverse correlation between temperature and effectiveness of CAR has been suggested in CA patients treated with TTM targeting either 33 °C, 36 °C or not treated with TTM [25]. Given the association between time and temperature in this study, it is not possible to infer whether the modifications in CAR are associated to the progression of brain damage or to the effect of temperature on brain homeostasis. No specific studies on the role of TTM on CAR in CA patients has been performed so far. However, a potential protective role of TTM on preserving the reactivity of brain vasculature after anoxic brain injury cannot be excluded. These findings should, therefore, increase the awareness of clinicians on the importance of an accurate management of temperature after rewarming from moderate hypothermia, as this might have an impact on cerebral perfusion. Future studies comparing patients with or without TTM after CA are necessary to understand the role of temperature on CAR in CA patients.

Regulation of CBF is a complex phenomenon, which depends on the interaction between metabolism and pressure regulation. Neuronal metabolism is probably the chief drive in modifying CBF, while the myogenic mechanism keeps CBF constant in case of modifications in cerebral perfusion pressure [35]. Data on CAR in case of altered metabolic demand are not univocal. Epileptic activity increases the diameter of the vessel supplying spiking neurons, thus increasing CBF [36]. Altered CAR has been documented in patients with seizures [37] and animal models showed persistent alteration in CAR after epileptic generalized activity [38]. On the opposite, CAR was intact in sedated patients with refractory status epilepticus [39] and during propofol-induced burst-suppression [40]; no specific data on CAR and burst-suppression secondary to severe brain damage has been reported so far. In our cohort, burst-suppression on EEG correlated with higher Mxa at NT. Whether altered CAR is a reflection of deranged metabolism or severe brain damage cannot be determined.

Major strengths of this study are the analysis of a relatively large cohort of patients and the use of a validated approach to assess CAR through spontaneous oscillations in MAP [41]. We systematically evaluated Mxa during HT and at NT, suggesting that time assessment is crucial to understand the role of CAR in CA patients. We should acknowledge also some limitations. We did not measure directly cerebrovascular resistance or absolute CBF values nor did we investigate MAP in relation to individual CAR curve. Cerebral perfusion after CA is probably heterogeneously deranged in the brain, with some areas more affected than others: since we insonated MCA, we cannot exclude that regional alterations in CAR may have gone undetected. Acute brain injury associated with CA may result in a narrowing of the pressure range for CAR, whose the lower limit may be observed at clinically accepted MAP of 60–70 mm Hg [7]. Therefore, we cannot exclude an overestimation of altered CAR in our patients due to the possibility of MAP falling outside the individual range of CAR. Altered CAR at NT could be secondary to an increase in intracranial pressure, which was not directly measured in this study. Cerebral autoregulation may be affected by several factors, such as modifications in the intracranial arteries diameter due to the effect of carbon dioxide arterial partial pressure. However, 45/50 (90%) of patients in our cohort had PaCO_2_ in the physiological range of 30–50 mmHg and no relationship was found between PaCO_2_ and Mxa in our cohort (Supplementary material). We defined altered CAR as Mxa > 0.3. However, such threshold has been used in previous literature and was based on the mathematical definition of moderate correlation. Indeed, Mxa is a continuous index, which likely reflects a continuum in cerebral autoregulation effectiveness [42, 43]. Finally, given the lack of published data on CAR in cardiac arrest patients, we could not properly calculate the sample size of this study and a convenient cohort of 50 patients was selected for this exploratory analysis. All patients who had CPC 5 at 3 months because of brain death or death by other cause were included in non-survivors and unfavourable outcome group. However, multi-organ failure was the cause of death in 8/31 patients (26%). Multi-organ failure refers to a condition, where more than one organ, system or apparatus is failing, including the brain. In such patients, diagnosis of brain death was not initiated due to the critical general condition. In addition, we only analyzed one fourth of CA patients admitted to our ICU, although most of exclusion criteria were reasonable, and the small sample size may bias statistical modeling. However, the possibility of small sample size bias and overfitting were limited by our modelling [44, 45]. Nevertheless, caution is advisable with regard to generalizability of our results, which should be confirmed in larger cohort.

## Conclusions

After TTM, cerebral autoregulation is frequently altered in CA during normothermia. Altered CAR during normothermia was independently associated with poor outcomes and other predictors of poor neurological recovery, such as highly malignant patterns on EEG and high NSE values.

## Supplementary Information

**Additional file 1:** Clinical and biological variables during targeted temperature management; details of outliers; Mxa values comparison among CPC groups; regularized regression models with elastic net; changes in Mxa according to the phase of temperature control; graphical representation of relative weight of tested variables in the predictive models.

## Data Availability

The data sets used and/or analysed during the current study are available from the corresponding author on reasonable request.

## Abbreviations

ABG: Arterial blood gases
BP: Arterial blood pressure
APACHE II: Acute physiologic assessment and chronic health evaluation
CA: Cardiac arrest
CAR: Cerebral autoregulation
CBF: Cerebral blood flow
cEEG: Continuous electroencephalography
CPC: Cerebral performance category
ECMO: Extra-corporeal membrane oxygenation
EEG: Electroencephalography
FiO_2_: Fraction of inspired oxygen
FO: Favourable outcome
FV: Blood flow velocity
HMp: Highly malignant EEG pattern
HT: Hypothermia
IABP: Intra-aortic balloon counter-pulsation
ICU: Intensive care unit
MAP: Mean systemic arterial pressure
MCA: Middle cerebral artery
Mxa: Mean flow index
NIRS: Near infrared spectroscopy
NMBA: Neuro-muscular blocking agent
NS: Non-survivors
NSE: Neuron-specific enolase
NT: Normothermia
PaCO2: Arterial carbon dioxide partial pressure
PaO_2_: Oxygen arterial partial pressure
PEEP: Positive end-expiratory pressure
ROSC: Return of spontaneous circulation
ScvO_2_: Central venous oxygen saturation
SU: Survivors
TBI: Traumatic brain injury
TCD: Transcranial Doppler
TTM: Targeted temperature management
UO: Unfavourable outcome
