# Supplementary material for: Cerebral autoregulation in anoxic brain injury patients treated with targeted temperature management
Source: J Intensive Care. 2021 Oct 26;9:67. doi: 10.1186/s40560-021-00579-z (PMC8547304; doi:10.1186/s40560-021-00579-z)
Supplement: Supplementary file 1 — Additional file 1: Clinical and biological variables during targeted temperature management; details of outliers; Mxa values comparison among CPC groups; regularized regression models with elastic net; changes in Mxa according to the phase of temperature control; graphical representation of relative weight of tested variables in the predictive models. [file 40560_2021_579_MOESM1_ESM.docx]

**Table 1. Clinical and biological variables during targeted temperature management** (TTM); HT = hypothermic phase; NT = normothermic phase. CAR cerebral autoregulation; FiO_2_ inspired fraction of oxygen; MAP mean arterial pressure; Mxa mean flow index; NMBA neuromuscular blocking agents; PEEP positive end-expiratory pressure; PaCO_2_ carbon dioxide arterial partial pressure; PaO_2_ oxygen arterial partial pressure; ScvO_2_ central venous oxygen saturation; TCD transcranial Doppler.

|  | **HT**  **(n=50)** | **NT**  **(n=50)** | **p-value** |
| --- | --- | --- | --- |
| Sedatives, n (%) | 50 (100) | 25 (50) | <0.01 |
| NMBA, n (%) | 41 (82) | 8 (16) | <0.01 |
| Mechanical ventilation, n (%) | 50 (100) | 34 (68) | <0.01 |
| MAP, mmHg | 76 [70 - 88] | 76 [70 - 82] | 0.93 |
| Heart rate, bpm | 68 [59 - 87] | 90 [74 - 107] | <0.01 |
| Temperature, °C | 33.7 [33.2 - 34] | 36.9 [36.6 - 37.4] | <0.01 |
| Hemoglobin, g/dL | 11.9 [9.8 – 13.5] | 11.6 [9.6 - 13.8] | 0.07 |
| PEEP, cmH_2_O | 6 [5 - 9] | 5 [5 - 8] | 0.98 |
| FiO_2_ | 0.30 [0.30 – 0.40] | 0.35 [0.30 – 0.45] | 0.36 |
| pH | 7.38 [7.34 - 7.42] | 7.36 [7.30 - 7.39] | 0.02 |
| PaCO_2_, mmHg | 39 [37 - 42] | 38 [35 - 40] | 0.22 |
| PaO_2_, mmHg | 82 [72 - 91] | 94 [80 - 105] | <0.01 |
| ScvO_2_, % | 76 [71 – 81] | 75 [72 – 78] | 0.26 |
| Lactate, mmol/L | 1.3 [1.0 - 2.2] | 1.6 [1.1 - 2.2] | 0.31 |
| Mxa | 0.33 [0.11 - 0.58] | 0.58 [0.30 - 0.83] | 0.03 |
| Intact CAR, n (%) | 23 (46) | 12 (24) | 0.03 |
| TCD assessment from admission, hours | 14 [9 – 18] | 38 [36 – 45] | <0.01 |
| Sedatives/analgesics, n (%)   - Morphine, mg/h - Midazolam, mg/h | 50 (100)  2 [0 – 3]  4 [3 – 6] | 25 (50)  0 [0 – 1]  0 [0 – 5] | <0.01  <0.01  0.01 |
| Cardio-active medications, n (%) | 34 (68) | 32 (64) | 0.83 |

**Table 2.** Details of outliers (n=4) identified during comparisons of Mxa between CPC groups.

|  | **Outliers**  **(n=19)** | **Others**  **(n=46)** |
| --- | --- | --- |
| **Male gender, n (%)** | 4 (100) | 39 (78) |
| **Age, years** | 60 [58-65] | 65 [55-73] |
| **APACHE II** | 29 [23-30] | 26 [24-31] |
| **ICU LOS, days** | 11 [7-20] | 9 [4-19] |
| **COMORBIDITIES** | | |
| **Arterial hypertension, n (%)** | 3 (75) | 23 (46) |
| **Vascular disease, n (%)** | 1 (25) | 10 (20) |
| **Chronic heart failure, n (%)** | 1 (25) | 3 (6) |
| **Chronic kidney disease, n (%)** | 0 (0) | 8 (16) |
| **COPD, n (%)** | 0 (0) | 6 (12) |
| **Diabetes mellitus, n (%)** | 0 (0) | 10 (20) |
| **Previous Seizure, n (%)** | 3 (75) | 3 (6) |
| **Minor stroke, n (%)** | 0 (0) | 7 (14) |
| **CARDIAC ARREST CHARACTERISTICS** | | |
| **Cause of CA, n (%)**   - *Cardiac* - *Anoxic* - *obstructive* - *Indeterminate* | 3 (75)  0 (0)  1 (25)  0 (0) | 23 (48)  15 (32)  2 (6)  9 (18) |
| **Shockable rhythm, n (%)** | 2 (50) | 24 (48) |
| **Time to ROSC, min** | 12 [10-16] | 20 [10-35] |
| **CLINICAL VARIABLES and AUTOREGULATION ASSESSMENT** | | |
| **NSE (ng/mL)** | na | 39 [24-78]  (n=32) |
| **Highly malignant EEG** | 0 (0) | 18 (36) |
| **Mxa during HT** | 0.32 [0.06-0.45] | 0.33  [0.11-0.58] |
| **Mxa during NT** | 0.92 [-0.07 – 0.95] | 0.58  [0.30-0.83] |
| **Altered CAR at HT, n (%)** | 2 (50) | 27 (54) |
| **Altered CAR at NT, n (%)** | 3 (75) | 38 (76) |

**Table 3.** Mxa values comparisons among CPC group excluding 3 outliers: two patients with arterial carbon dioxide partial pressure > 50 mmHg and one patient who died because of MOF on high dose of cardio-active medications.

|  | Sum of Squares | df | Mean Square | F | Sig. |
| --- | --- | --- | --- | --- | --- |
| Between Groups | 2.411 | 3 | .804 | 13.495 | .000 |
| Within Groups | 2.442 | 41 | .060 |  |  |
| Total | 4.854 | 44 |  |  |  |

| **Multiple Comparisons** | | | | | | |
| --- | --- | --- | --- | --- | --- | --- |
| Dependent Variable: Mxa at NT | | | | | | |
| Tukey HSD | | | | | | |
| (I) CPC | (J) CPC | Mean Difference (I-J) | Std Error | Sig. | 95% Confidence interval | |
|  |  |  |  |  | Lower Bound | Upper Bound |
| 1 | 2 | .03767 | .14778 | .994 | -.3580 | .4334 |
|  | 3 | -.13833 | .14778 | .786 | -.5340 | .2574 |
|  | 5 | -.50626^*^ | .10946 | .000 | -.7994 | -.2132 |
| 2 | 1 | -.03767 | .14778 | .994 | -.4334 | .3580 |
|  | 3 | -.17600 | .15435 | .667 | -.5893 | .2373 |
|  | 5 | -.54393^*^ | .11818 | .000 | -.8604 | -.2275 |
| 3 | 1 | .13833 | .14778 | .786 | -.2574 | .5340 |
|  | 2 | .17600 | .15435 | .667 | -.2373 | .5893 |
|  | 5 | -.36793^*^ | .11818 | .017 | -.6844 | -.0515 |
| 5 | 1 | .50626^*^ | .10946 | .000 | .2132 | .7994 |
|  | 2 | .54393^*^ | .11818 | .000 | .2275 | .8604 |
|  | 3 | .36793^*^ | .11818 | .017 | .0515 | .6844 |
| *The mean difference is significant at the 0.05 level | | | | | | |

**Table 4. Regularized regression models with elastic net**. The fitted models showed an accuracy of 86% (CI 73-94%), a sensitivity 97% and a specificity 68% for mortality, and an accuracy 84% (CI 71-93%), a sensitivity 100% and a specificity 43% to predict unfavorable neurological outcome (UO). EEG HMp highly malignant electroencephalography during ICU stay; Mxa mean flow index during normothermia; OR odds ratio; pCO_2_ arterial carbon dioxide partial pressure during normothermia; ROSC return of spontaneous circulation; cardiac medications: noradrenaline (norepinephrine) and/or dobutamine.

^*^ The parameter alpha has been selected according to the minimization of the partial likelihood deviance of the model; the lambda parameter was determined using grid search with 10-fold cross-validation and the optimal value was determined by minimizing the deviance of the model selected.

|  | **Mortality** | **UO** |
| --- | --- | --- |
|  | **Coefficient** | **Coefficient** |
| (Intercept) | 0.570 | 1.113 |
| Age | 0.041 | 0.085 |
| Arterial hypertension | 0.031 | 0.106 |
| Shockable rhythm | -0.283 | -0.229 |
| Time to ROSC | 0.017 | 0.113 |
| Cardiac medications | 0.120 | 0.133 |
| PaCO_2_ | -0.135 | -0.208 |
| EEG HMp | 0.463 | 0.431 |
| Mxa | 0.295 | 0.298 |
| (alpha; lambda)^*^ parameter | (0.2; 0.247) | (0.1; 0.182) |

**Figure 1.** **Changes in Mxa, according to the phase of temperature control** (HT = hypothermia; NT = normothermia) and the measured outcomes (FO = favorable neurological outcome; UO = unfavorable neurological outcome).


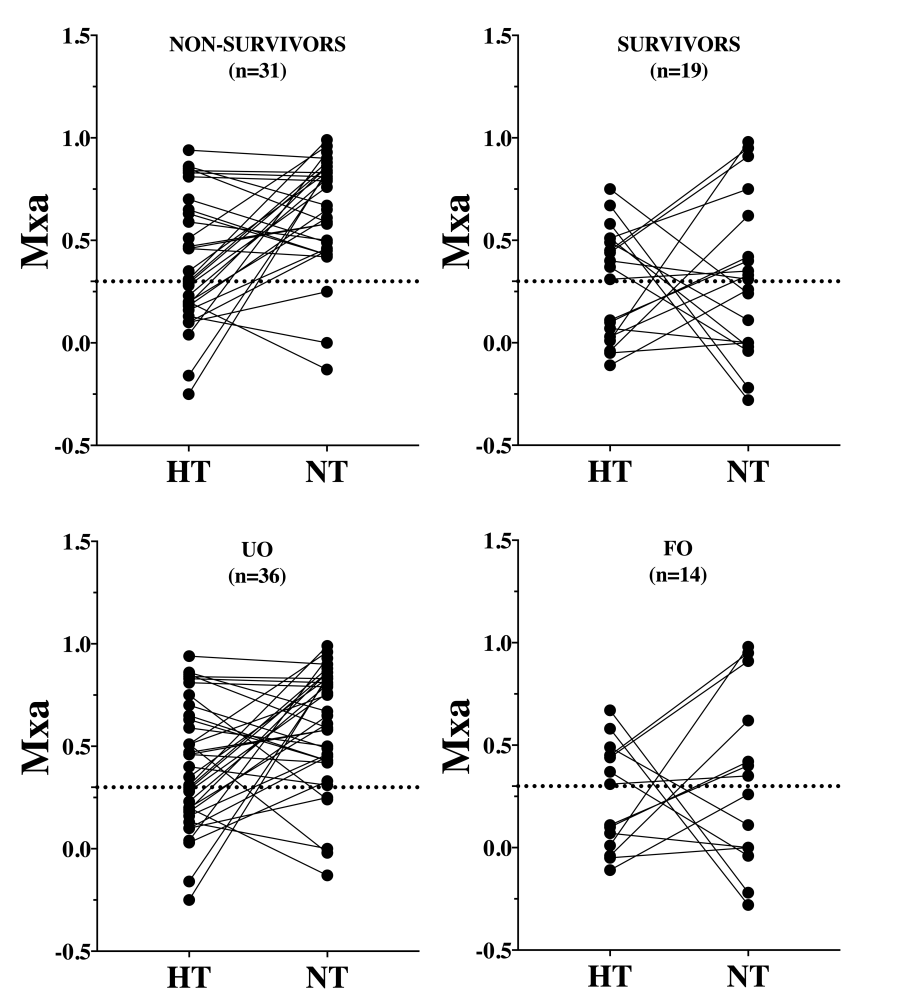


**Figure 2. Graphical representation of relative weight of tested variables in the predictive models.** EEG HMp highly malignant electroencephalography during ICU stay; Mxa mean flow index during normothermia; PaCO_2_ arterial carbon dioxide partial pressure during normothermia; ROSC return of spontaneous circulation; cardiac medications: noradrenaline (norepinephrine) and/or dobutamine.


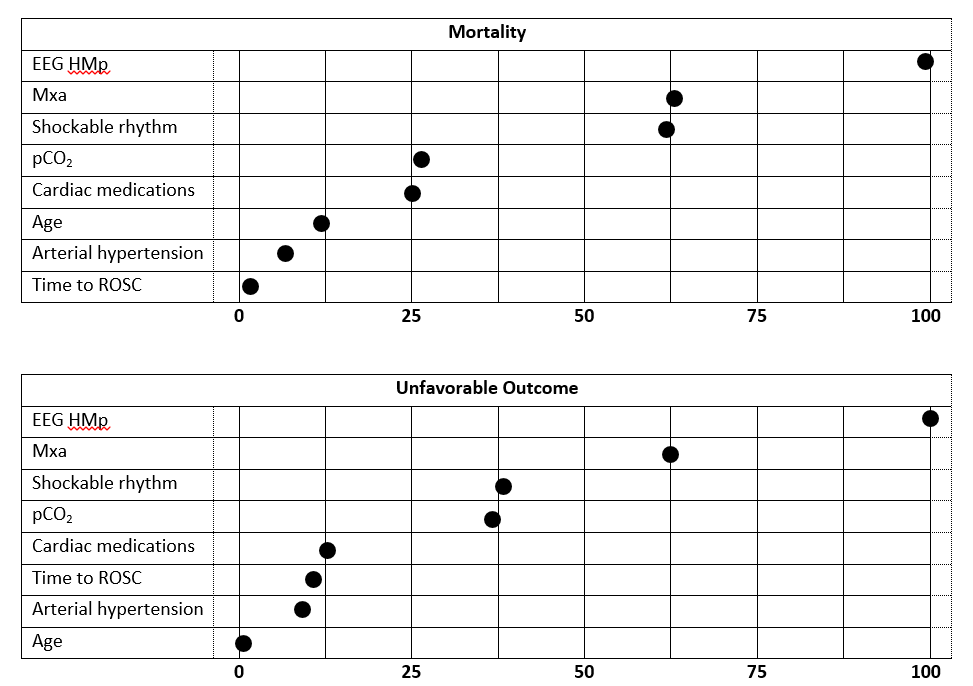


**Fig 3.** Scatter plot of Mxa vs PaCO2 values.
